# Supplementary material for: DNA binding analysis of rare variants in homeodomains reveals homeodomain specificity-determining residues
Source: Nat Commun. 2024 Apr 10;15:3110. doi: 10.1038/s41467-024-47396-0 (PMC11006913; doi:10.1038/s41467-024-47396-0)
Supplement: Supplementary file 1 — Supplementary Information [file 41467_2024_47396_MOESM1_ESM.pdf]

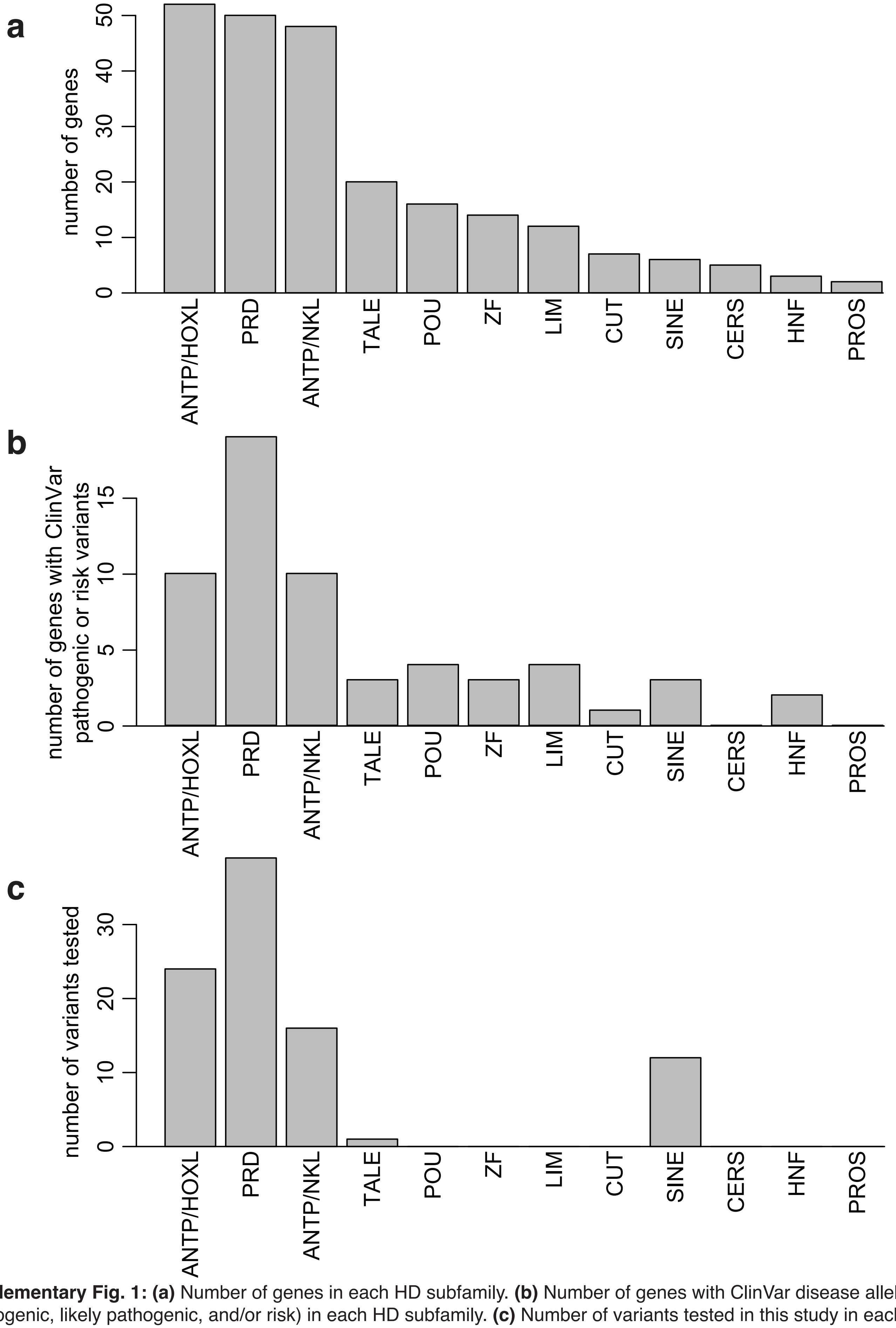

Supplementary Fig. 1: (a) Number of genes in each HD subfamily. **(b)** Number of genes with ClinVar disease alleles (pathogenic, likely pathogenic, and/or risk) in each HD subfamily. **(c)** Number of variants tested in this study in each HD subfamily. Source data are provided as a Source Data file.[37]

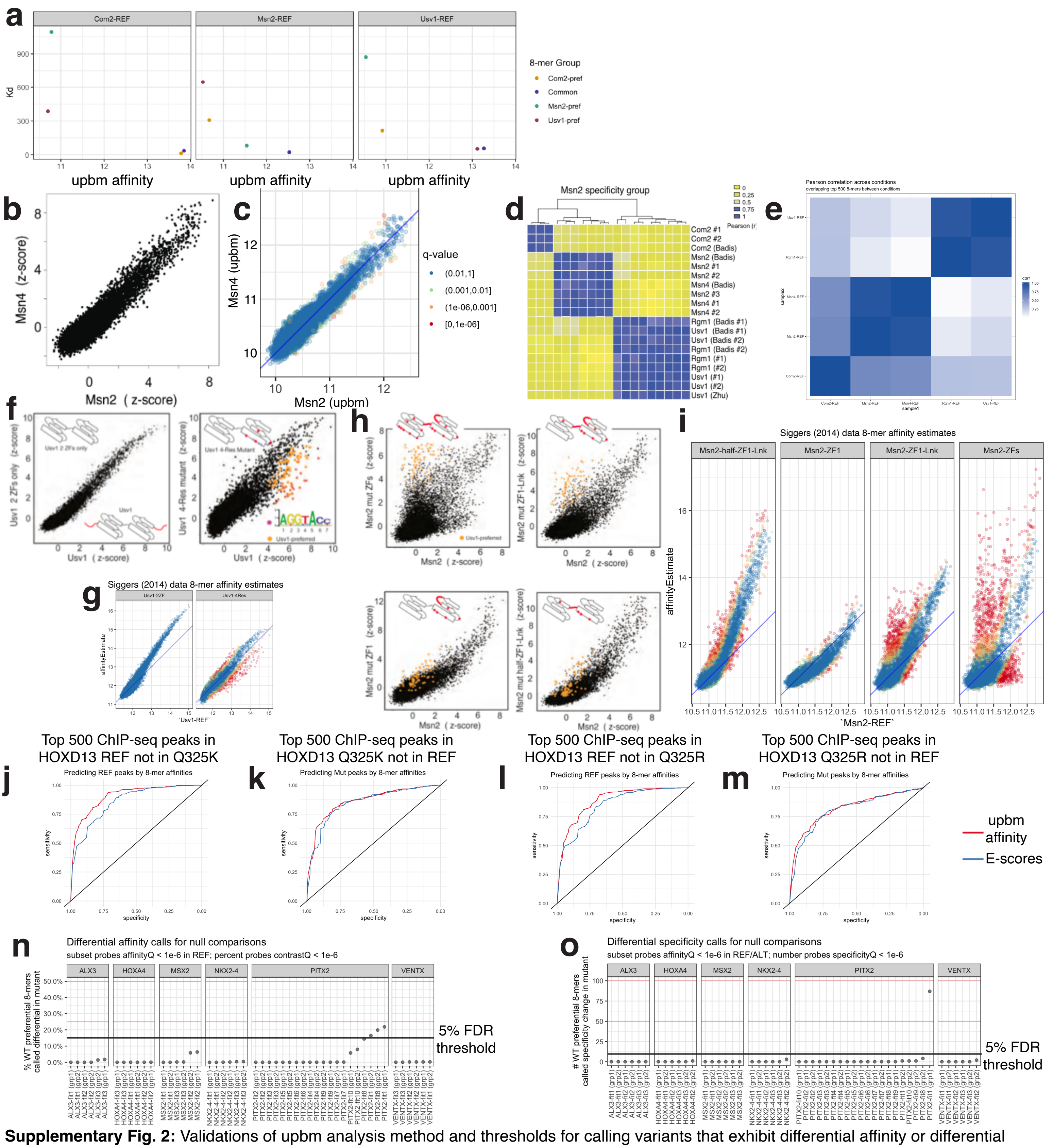

Supplementary Fig. 2: Validations of upbm analysis method and thresholds for calling variants that exhibit differential affinity or differential specificity. **(a)** upbm affinity scores accurately discriminate 8-mers bound preferentially by Com2 (left), Msn2 (middle), or Usv1 (right) or by all 3 proteins, as compared to 8-mers bound preferentially by either of the other 2 proteins, based on previously published PBM data analyzed by a prior analysis method ("8-mer group") and Kd data derived from EMSAs [36]. **(b)** Previously reported identical binding of Msn2 and Msn4 over all 8-mers [36] is **(c)** recapitulated by upbm 8-mer affinity scores. **(d)** The binding profiles of the top 500 bound 8-mers bound by Msn2 or Msn4 being identical and distinct from those of the Rgm1 and Usv1 paralogs and Com2, as reported previously [36], is **(e)** recapitulated by the top 500 bound 8-mers based on upbm affinity scores. Previously reported effects of mutations in **(f)** Usv1 and **(h)** Msn2 [36] are recapitulated by upbm 8-mer affinity estimates derived from the PBM data for **(g)** Usv1 and **(i)** Msn2. **(j-m)** upbm affinity scores for HOXD13 **(j, l)** reference, **(k)** Q325K, or **(m)** Q325R accurately discriminated HOXD13 ChIP-Seq peaks bound preferentially by the respective ectopically expressed HOXD13 variant (reference or mutant) [17]. **(n)** Fraction of significantly bound 8-mers with significantly altered affinity in null (reference vs. reference) comparisons and selection of 5% FDR threshold for calling affinity-changing variants. **(o)** Number of significantly bound 8-mers with significant specificity in null (reference vs. reference) comparisons and selection of 5% FDR threshold for calling specificity-changing variants. Source data are provided as a Source Data file.[37]

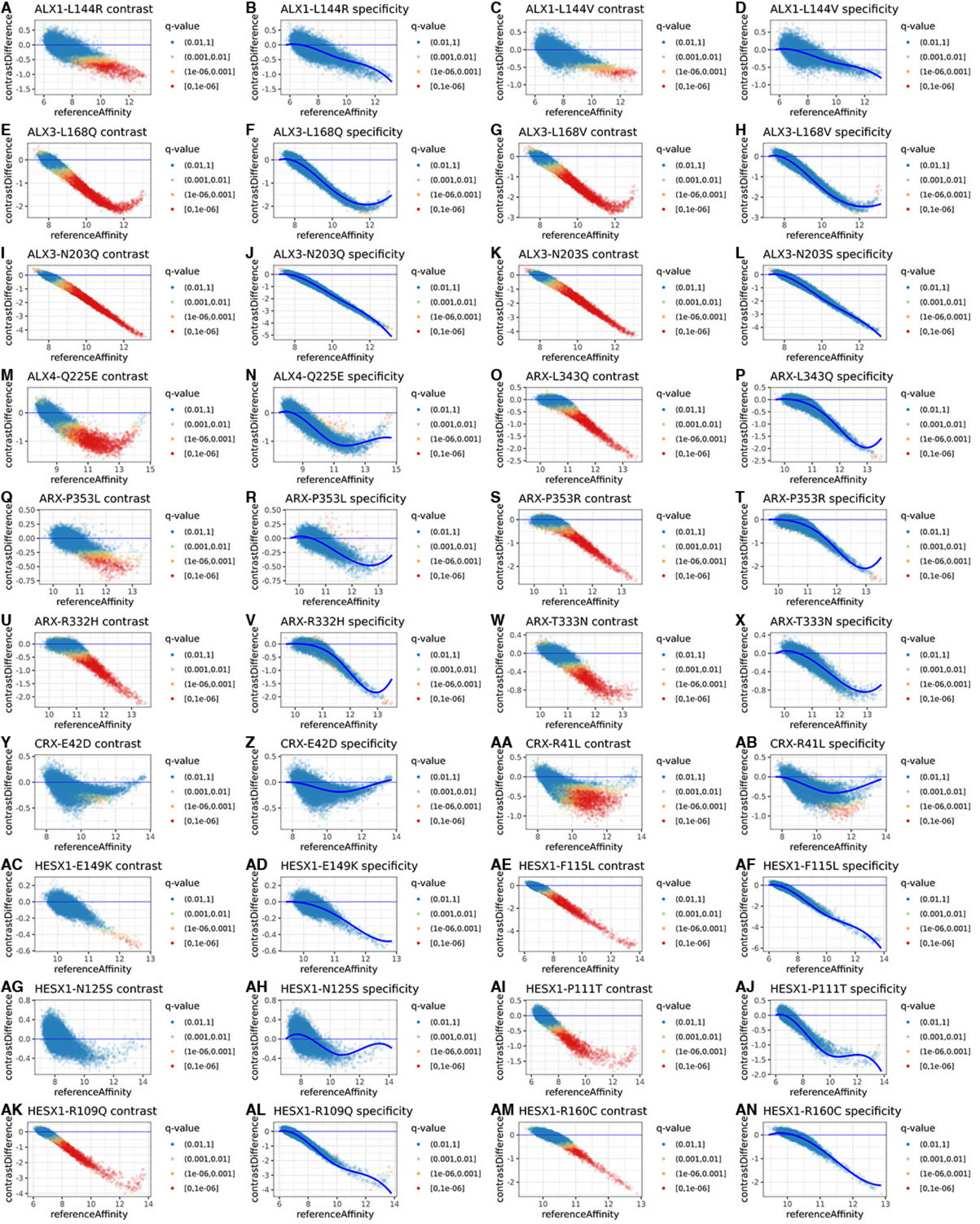

Supplementary Fig. 3: Differential 8-mer MA plots colored by contrastQ (left) and specificityQ (right) for all variants. Source data are provided as a Source Data file.[37]

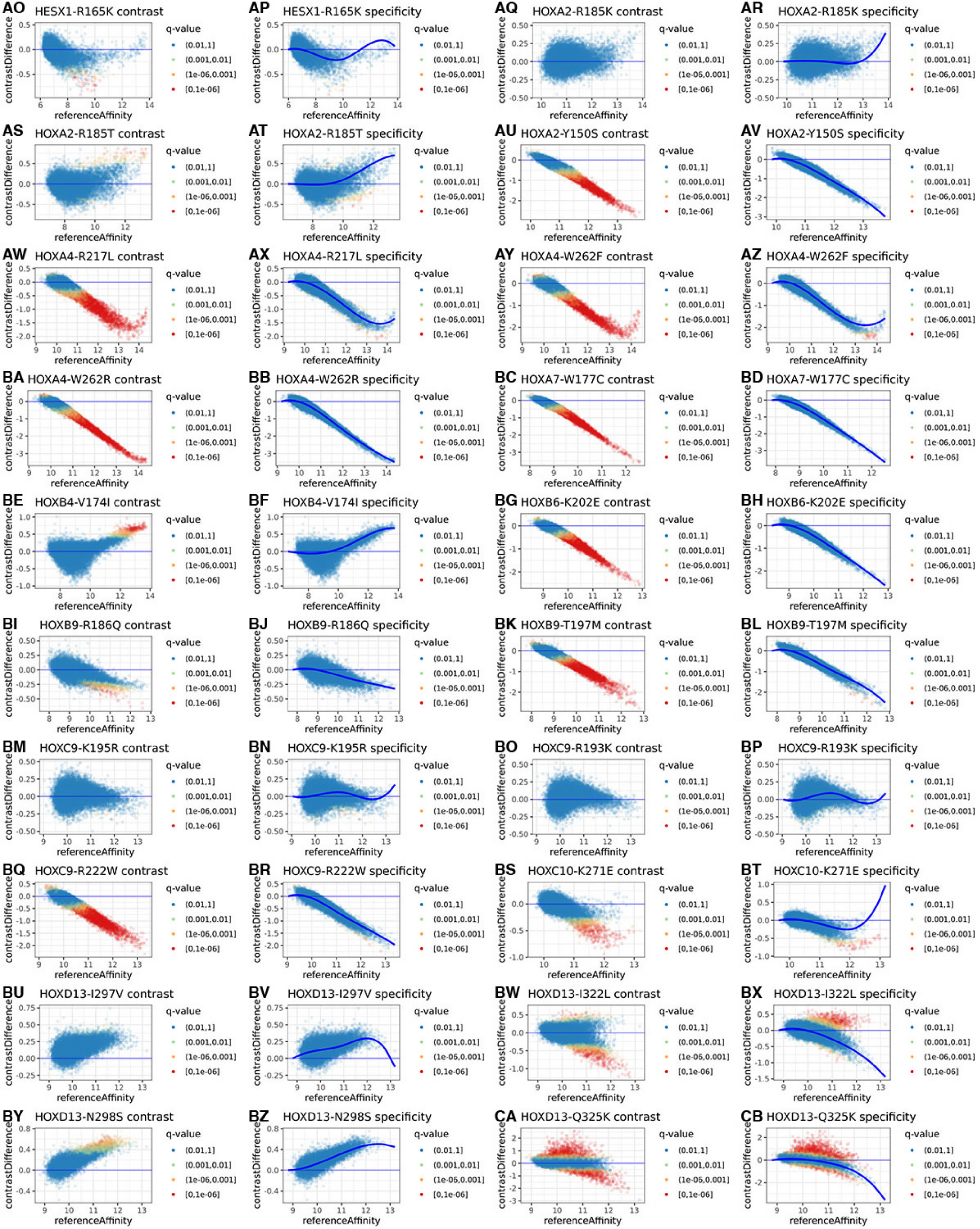

Supplementary Fig. 3, continued.

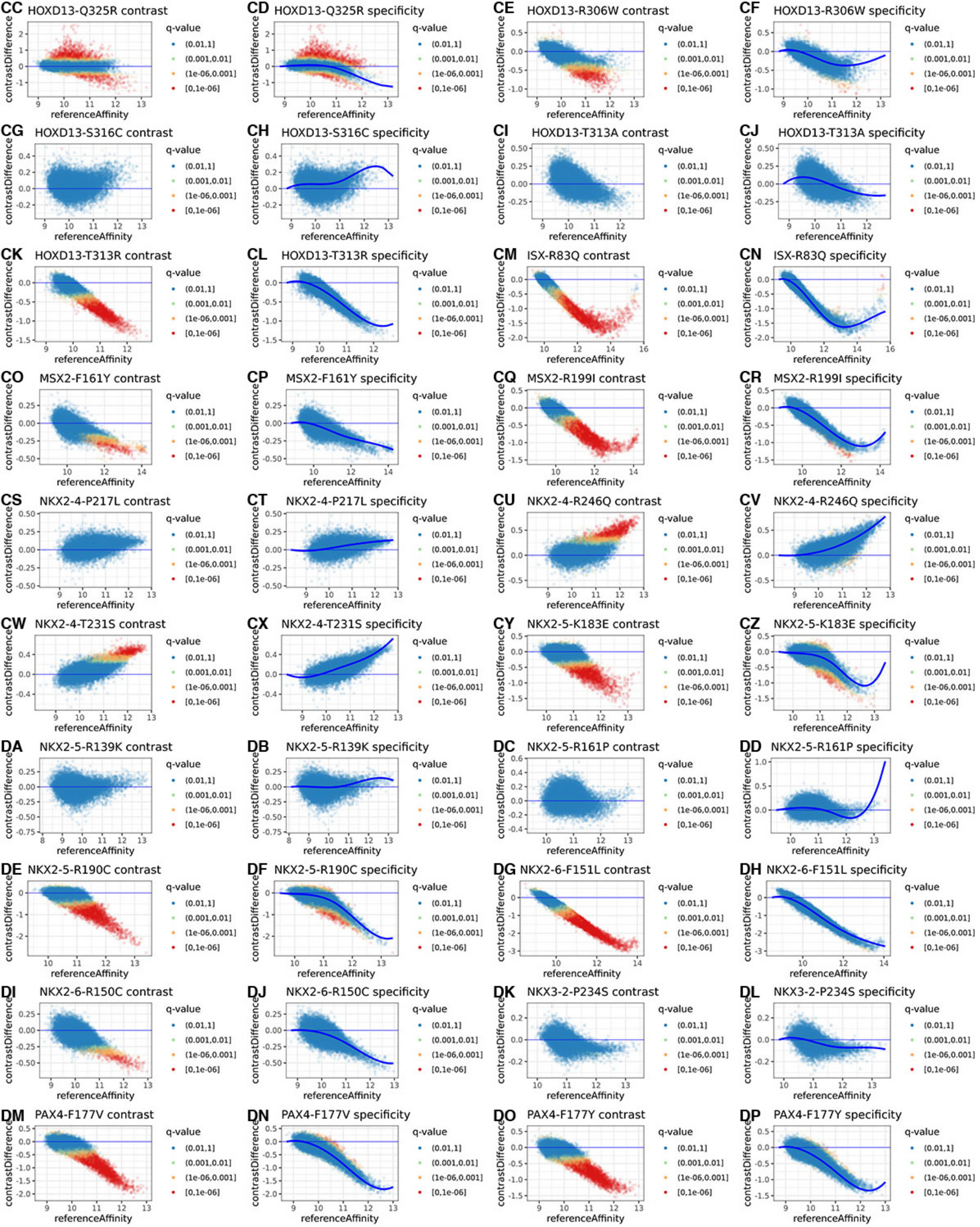

Supplementary Fig. 3, continued.

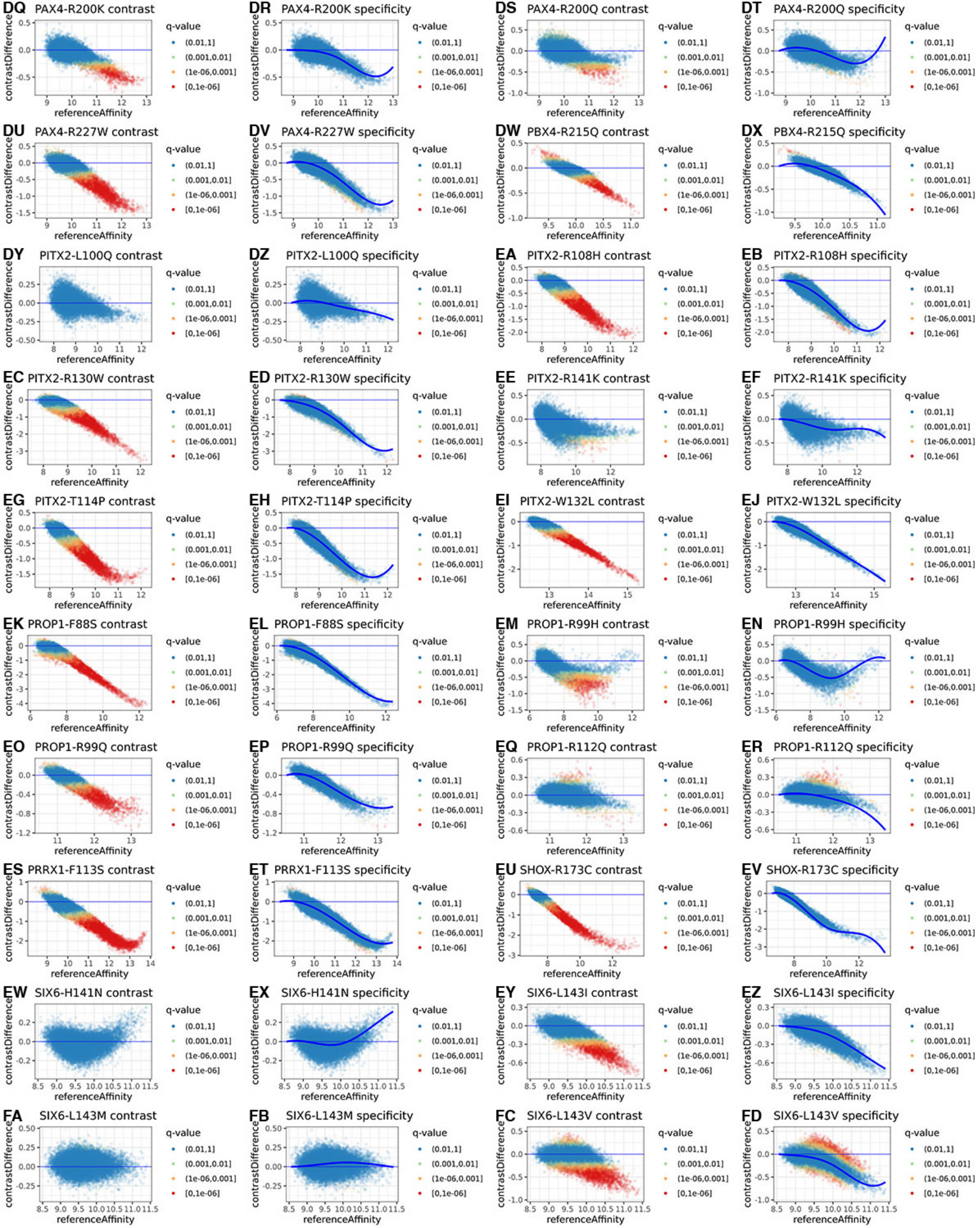

Supplementary Fig. 3, continued.

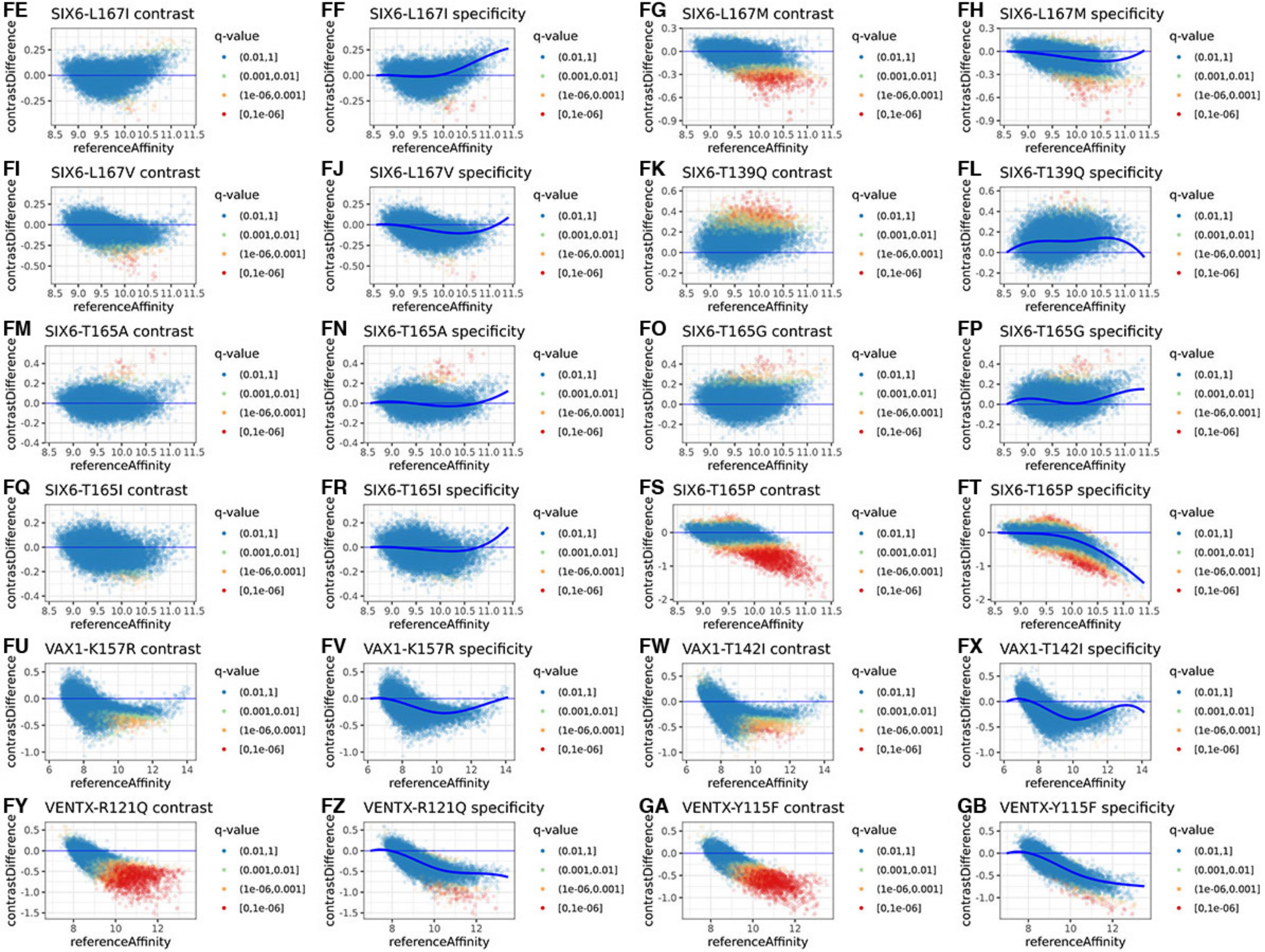

Supplementary Fig. 3, continued.

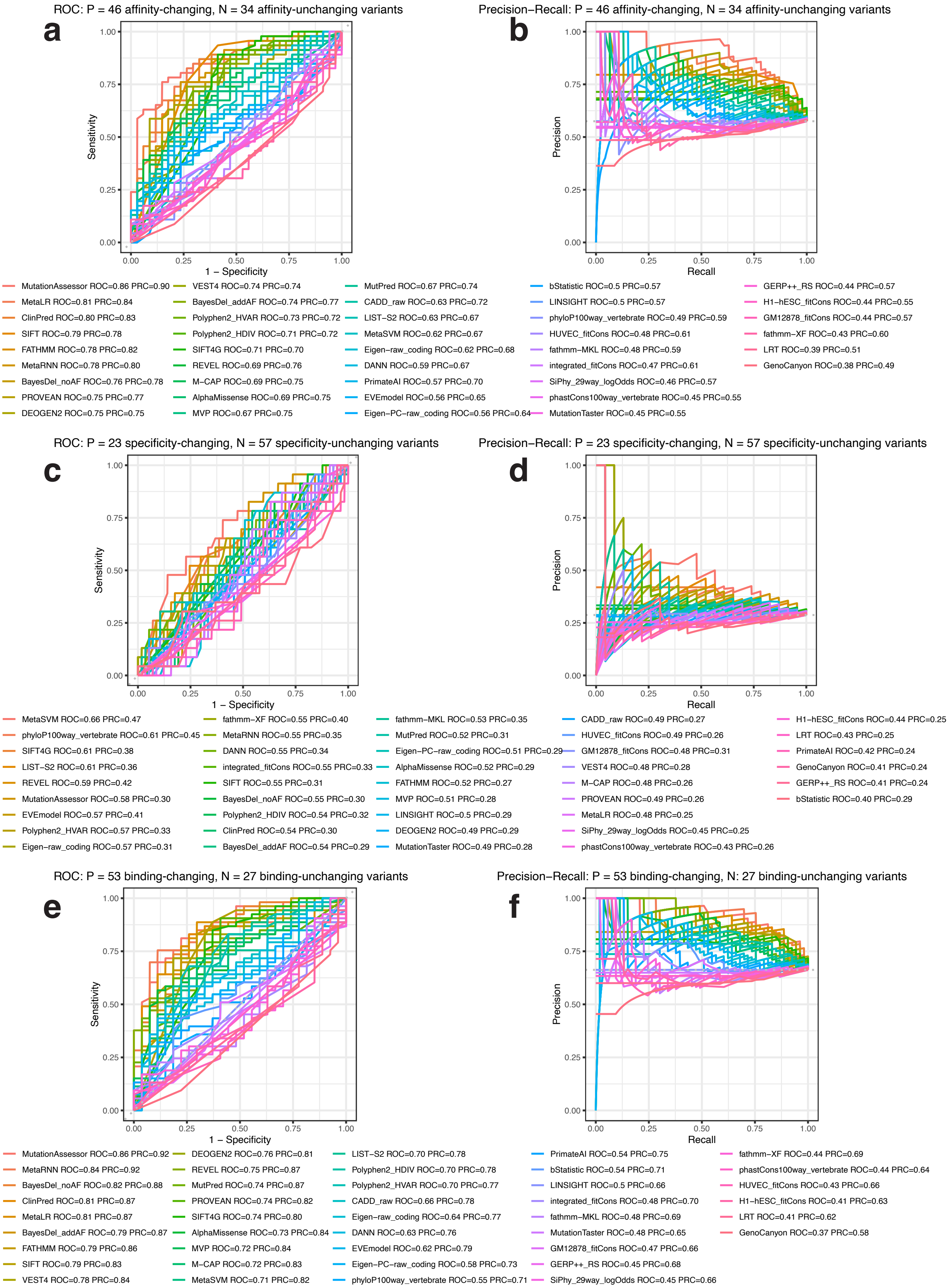

Supplementary Fig. 4: AUROC and AUPRC plots for all variant effect prediction tools for discriminating variants with altered **(a,b)** affinity, (**c,d**) specificity, or (**e,f)** binding (*i.e.,* affinity and/or specificity). Source data are provided as a Source Data file.[37]

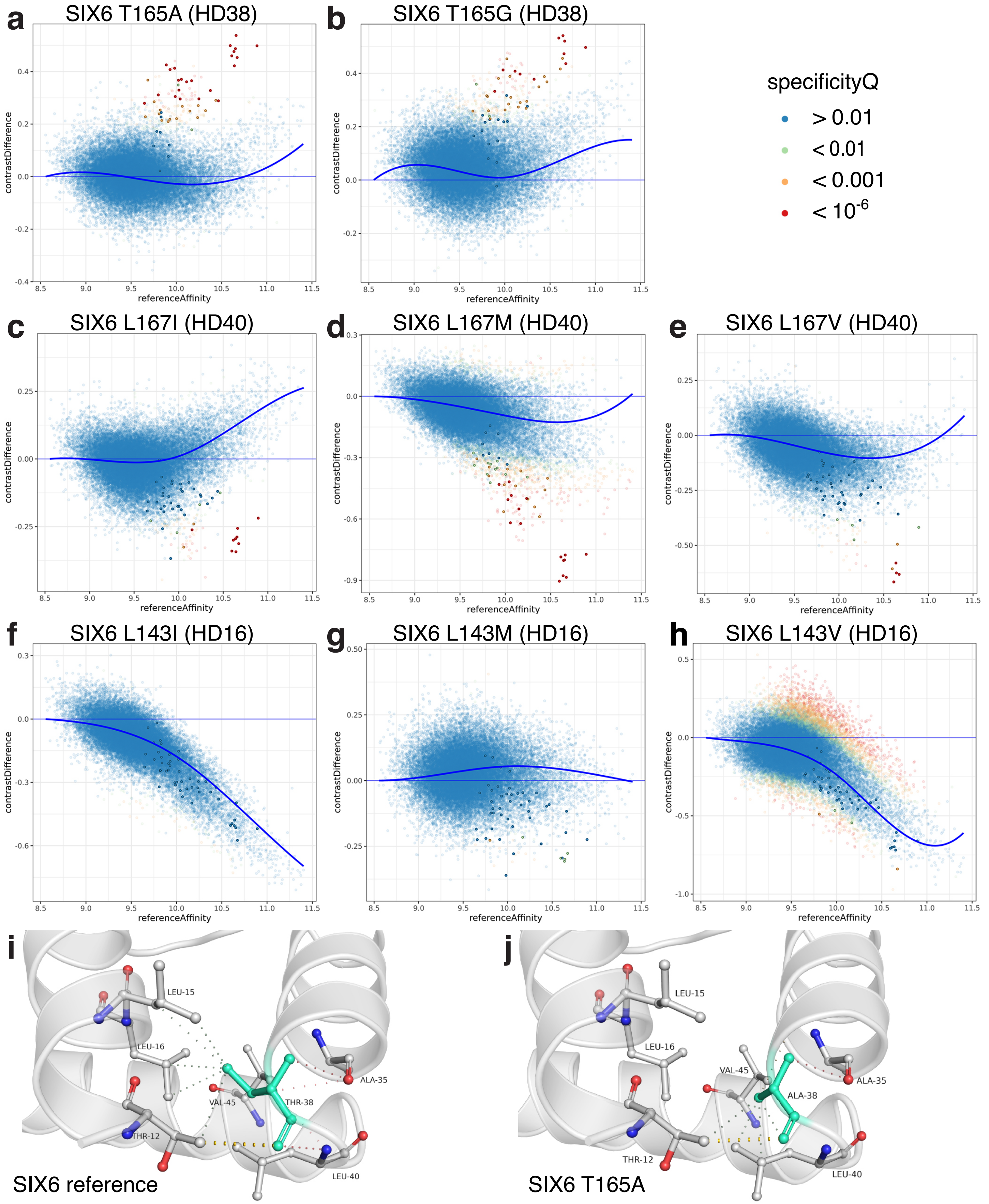

Supplementary Fig. 5: Amino acid positions distal to the DNA-binding interface affect specificity in SIX6. The **(a)** T165A and **(b)** T165G variants preferentially increase affinity for 8-mers containing a TGACAC motif (highlighted points). The **(c)** L167I, **(d)** L167M, and **(e)** L167V variants preferentially decrease binding to the same 8-mers. The **(f)** L143I, **(g)** L143M, and **(h)** L143V variants have more variable effects. **(i,j)** Homology modeling suggests that a network of contacts including HD positions 16, 38, and 40 (L143, T165, and L167 in SIX6) in **(i)** the reference allele is **(j)** disrupted in the T165A variant. Source Data are provided as a Source Data file.[37]

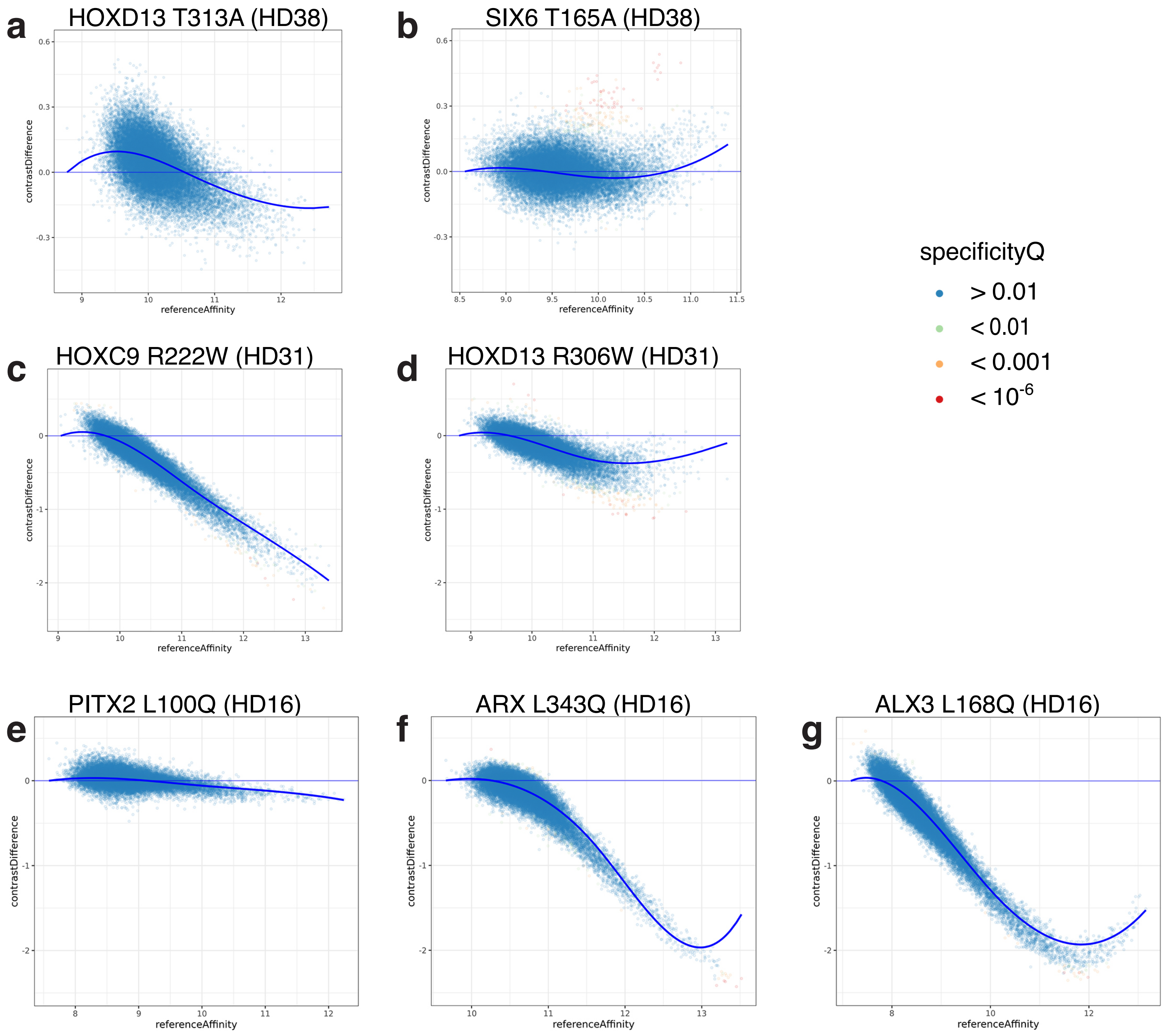

Supplementary Fig. 6: Same substitution in different HDs showed different effects on DNA binding activity. **(a)** Thr to Ala substitution at HD canonical position 38 resulted in no change in DNA binding activity in HOXD13, but **(b)** altered specificity in SIX6. **(c)** Arg to Trp substitution at canonical position 31 resulted in strongly reduced affinity in HOXC9, an ANTP/HOXL HD, but **(d)** mildly reduced affinity and altered specificity in HOXD13, another ANTP/HOXL HD. **(e)** Leu to Gln substitution at position 16 showed no effect in PITX2, but resulted in strongly decreased affinity in **(f)** ARX and **(g)** ALX3. Source data are provided as a Source Data file.[37]

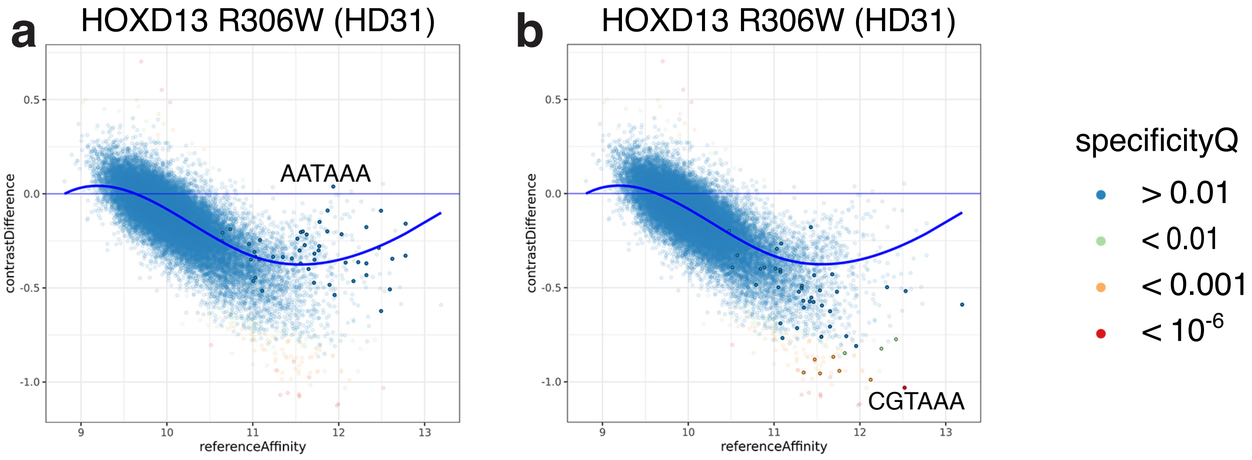

Supplementary Fig. 7: HOXD13 R306W variant appears to differentially affect binding to **(a)** AATAAA- vs. **(b)** CGTAAA-containing 8mers. Source data are provided as a Source Data file.[37]

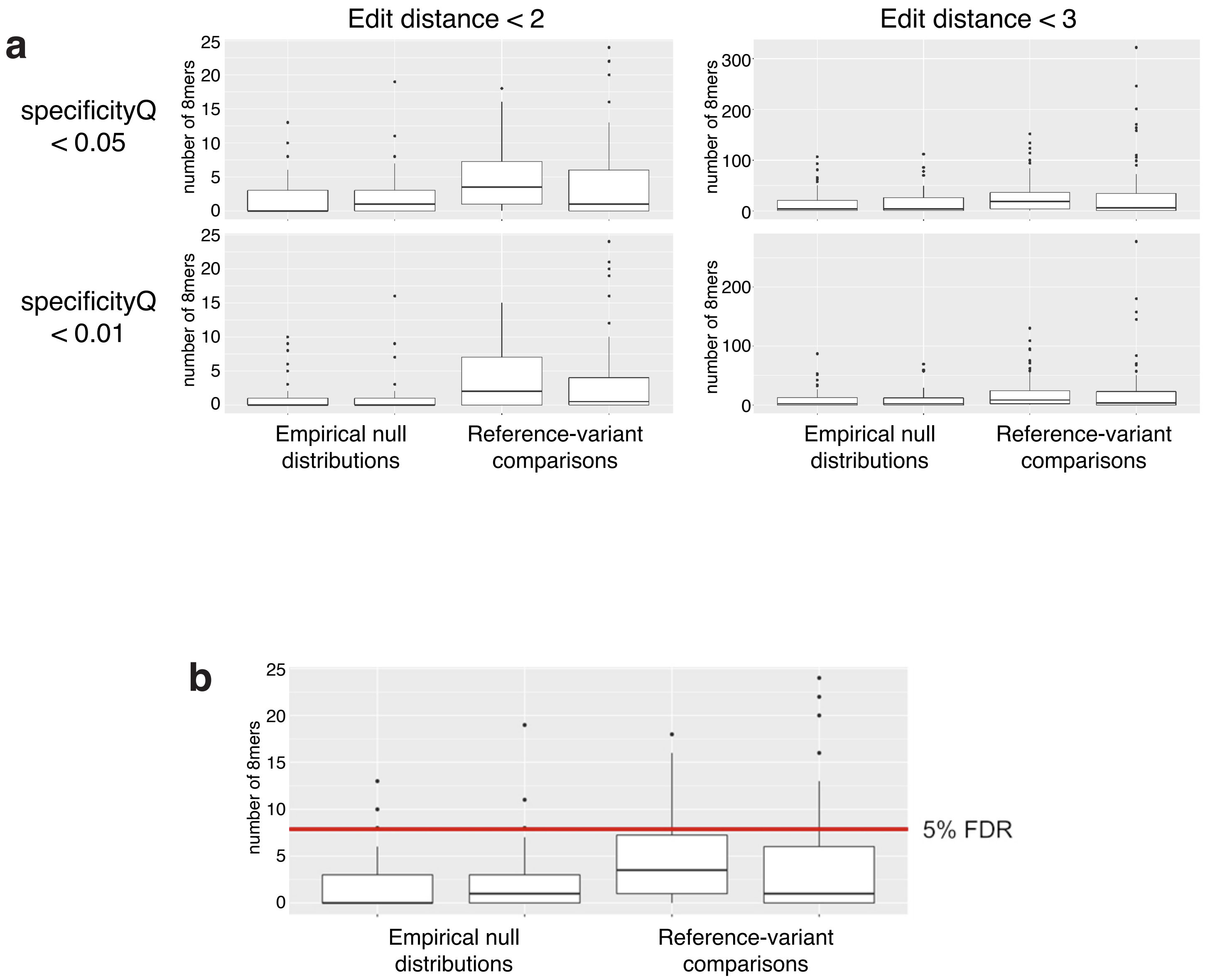

Supplementary Fig. 8: (a) Distribution of numbers of 8-mers with specificityQ below threshold within an edit distance below threshold of the top differentially bound 8-mer, for various thresholds, in null (reference vs. reference) comparisons (left) or variant vs. reference comparisons (right). **(b)** Example of the choice of a 5% FDR threshold for calling specificity-altering variants, in this case for specificityQ < 0.05 and edit distance < 2. Heavy lines represent median values, box limits upper and lower quartiles, whiskers 1.5 x interquartile range, and points show individual values outside this range. Source data are provided as a Source Data file.[37]

Supplementary Table 1: Published DNA binding assays of HD variants tested in this study and their results.

variant	probe.seq	max8mer	contrastDifference	contrastQ	binding.result	ref
ARX-L343Q	GTAATGAATTGATTTAATTAACAGGGGAGTCTGA	GTTAATTA	-2.292847	3.05E-194	total loss of binding	PMID: 22252899
ARX-L343Q	GTAATGAATTGATTTAATTAACAGGGGAGTCTGA	GTTAATTA	-2.292847	3.05E-194	total loss of binding	PMID: 22194193
ARX-P353L	GTAATGAATTGATTTAATTAACAGGGGAGTCTGA	GTTAATTA	-0.3896133	1.83E-05	moderate reduction of binding	PMID: 22194193
ARX-P353R	GTAATGAATTGATTTAATTAACAGGGGAGTCTGA	GTTAATTA	-2.415833	1.40E-220	moderate reduction of binding	PMID: 22252899
ARX-P353R	GTAATGAATTGATTTAATTAACAGGGGAGTCTGA	GTTAATTA	-2.415833	1.40E-220	total loss of binding	PMID: 22194193
ARX-T333N	GTAATGAATTGATTTAATTAACAGGGGAGTCTGA	GTTAATTA	-0.8031877	5.30E-28	moderate reduction of binding	PMID: 22252899
ARX-T333N	GTAATGAATTGATTTAATTAACAGGGGAGTCTGA	GTTAATTA	-0.8031877	5.30E-28	total loss of binding	PMID: 22194193
HESX1-E149K	AGCTTGAGTCTAATTGAATTACTGTAC	CTAATTGA	-0.5021853	9.83E-08	moderate reduction of binding	PMID: 17148560
HESX1-N125S	AGCTTGAGTCTAATTGAATTACTGTAC	CTAATTGA	-0.1720494	0.9069244	increase of binding	PMID: 11748154
HESX1-N125S	TGCAATTAAATTCCCAGGATTATCAT	CAATTAAA	-0.2768801	0.7783541	increase of binding	PMID: 11748154
HESX1-R109Q	AGCTTGAGTCTAATTGAATTACTGTAC	CTAATTGA	-3.401095	1.24E-92	strong loss of binding	PMID: 26781211
HESX1-R160C	AGCTTGAGTCTAATTGAATTACTGTAC	CTAATTGA	-2.104143	1.53E-116	total loss of binding	PMID: 11748154
HESX1-R160C	AGCTTGAGTCTAATTGAATTACTGTAC	CTAATTGA	-2.104143	1.53E-116	total loss of binding	PMID: 9620767
HOXD13-I322L	GGGATCTGACAGTTTTACGACAGATCT	TCGTAAAA	0.2591863	3.41E-01	increase of binding	PMID: 12649808
HOXD13-I322L	GGGATCTGACAGTTTTATGACAGATCT	TCATAAAA	-1.149515	8.96E-16	moderate reduction of binding	PMID: 12649808
HOXD13-T313R	GGATCCCAATAAAATCGGC	CAATAAAA	-1.234638	1.85E-52	strong loss of binding	PMID: 26581570
HOXD13-S316C	GGGATCTGACAGTTTTACGACAGATCT	TCGTAAAA	0.127865	0.9698003	increase of binding	PMID: 12649808
HOXD13-S316C	GGGATCTGACAGTTTTATGACAGATCT	TCATAAAA	0.132662	0.9698003	slight reduction of binding	PMID: 12649808
NKX2-5-K183E	TCAAGTGCACTTGATCAAGTGCACTTGA	GCACTTGA	-1.437424	1.87E-56	total loss of binding	PMID: 15917268
NKX2-5-K183E	TGAAGTGCTCTTGATGAAGTGCTCTTGATGAAGTG[illegible]	GCACTTCA	-1.27106	5.13E-38	total loss of binding	PMID: 15917268
PROP1-F88S	ACTAATTGAATTAGC	CTAATTGA	-4.12405	3.19E-277	total loss of binding	PMID: 10946881
PROP1-F88S	ACTAATTGAATTAGC	CTAATTGA	-4.12405	3.19E-277	total loss of binding	PMID: 12519826
PROP1-R99Q	ACTAATTGAATTAGC	CTAATTGA	-0.6870554	2.52E-38	strong loss of binding	PMID: 12519826
SHOX-R173C	TAATGGCATTATAATGGCATTATAATGGCATTA	ATTATAAT	-1.910817	1.38E-15	strong loss of binding	PMID: 15931687

Supplementary Table 2: PDB template structures used to model TFs in this study.

Gene	HD subfamily of gene	PDB template	% seq. identity	PDB: gene	PDB: Organism	PDB: HD subfam	PDB: DNA template	kmer modeled	PDB: refs
**ALX1**	PRD	3A01:B	68.66	AL	Fruit fly	PRD-like	GGCTTAATTAATTGCGG	CTAATTAG	PMID: 20389279
**ALX3**	PRD	3A01:B	68.66	AL	Fruit fly	PRD-like	GGCTTAATTAATTGCGG	ATTAATTA	PMID: 20389279
**ALX4**	PRD	3A01:B	71.64	AL	Fruit fly	PRD-like	CCGCAATTAATTAAGCC	ATTAATTA	PMID: 20389279
**ARX**	PRD	3A01:B	84.62	AL	Fruit fly	PRD-like	CCGCAATTAATTAAGCC	CTAATTAG	PMID: 20389279
**CRX**	PRD	3CMY:A	98.36	PAX3	Human	PRD	TGTAATCGATTATG	ATAATCCG	PMID: 19199574
**HESX1**	PRD	1FJL:A	43.21	PRD	Fruit fly	PRD	TGTAATCAGATTAT	TTAATTAA	PMID: 7671301
**HOXA2**	ANTP/HOXL	2H1K:A	66.67	PDX1	Golden hamster	ANTP/HOXL	TCTCTAATGAGTTTC	GTAATTAC	PMID: 17315980
**HOXA4**	ANTP/HOXL	2R5Z:A	69.32	SCR	Fruit fly	ANTP/HOXL	TCAGCCGATTAATCTTAGAG	ATTAATTA	PMID: 17981120
**HOXA7**	ANTP/HOXL	9ANT:A	93.55	ANTP	Fruit fly	ANTP/HOXL	TCTCTAATGGCTTC	ATTAATTA	PMID: 9699632
**HOXB4**	ANTP/HOXL	9ANT:A	79.03	ANTP	Fruit fly	ANTP/HOXL	TCTCTAATGGCTTC	ATTAATTA	PMID: 9699632
**HOXB6**	ANTP/HOXL	4XIC:A	90.16	ANTP	Fruit fly	ANTP/HOXL	TCTCTAATGGCTTTC	GGTAATTA	PMID: 26331260
**HOXB9**	ANTP/HOXL	1PUF:A	87.01	HOXA9	Mouse	ANTP/HOXL	TAGCGTCGTAAATCATAGAG	CCATAAAA	PMID: 12923056
**HOXC10**	ANTP/HOXL	1PUF:A	71.43	HOXA9	Mouse	ANTP/HOXL	TAGCGTCGTAAATCATAGAG	GTCGTAAA	PMID: 12923056
**HOXC9**	ANTP/HOXL	1PUF:A	87.01	HOXA9	Mouse	ANTP/HOXL	TAGCGTCGTAAATCATAGAG	CCATAAAA	PMID: 12923056
**HOXD13**	ANTP/HOXL	5EDN:A	70.67	HOXB13	Human	ANTP/HOXL	GGACCTCGTAAAACACAAC	CTCGTAAA	PMID: 29638214
**ISX**	PRD	3A01:B	82.09	AL	Fruit fly	PRD-like	CCGCAATTAATTAAGCC	CTAATTAC	PMID: 20389279
**MSX2**	ANTP/NKL	1IG7:A	96.55	MSX1	Mouse	ANTP/NKL	TCCTTCAATTAGTGAC	GCTAATTA	PMID: 11580277
**NKX2-4**	ANTP/NKL	5FLV:E	40.74	NKX2-5	Mouse	ANTP/NKL	TCTTCTCACACCTTTGAAGTGG	CCACTTGA	PMID: 26875865
**NKX2-5**	ANTP/NKL	3RKQ:A	100	NKX2-5	Human	ANTP/NKL	TCAAGAGGCCCCCACTTCA	CCACTTGA	PMID: 22849347
**NKX2-6**	ANTP/NKL	3RKQ:A	86.21	NKX2-5	Human	ANTP/NKL	TCAAGAGGCCCCCACTTCA	CCACTTAA	PMID: 22849347
**NKX3-2**	ANTP/NKL	5FLV:E	68.97	NKX2-5	Mouse	ANTP/NKL	TCTTCTCACACCTTTGAAGTGG	CCACTTAG	PMID: 26875865
**PAX4**	PRD	1FJL:A	41.25	PRD	Fruit fly	PRD	TGTAATCAGATTAT	CTAATTAG	PMID: 7671301
**PBX4**	TALE	1PUF:B	87.67	PBX1	Human	TALE	TAGCGTCGTAAATCATAGAG	ACATGTCA	PMID: 12923056
**PITX2**	PRD	3A01:B	61.19	AL	Fruit fly	PRD-like	CCGCAATTAATTAAGCC	GGGATTAA	PMID: 20389279
**PROP1**	PRD	3A01:B	61.19	AL	Fruit fly	PRD-like	CCGCAATTAATTAAGCC	CTAATTAG	PMID: 20389279
**PRRX1**	PRD	3A01:B	65.67	AL	Fruit fly	PRD-like	CCGCAATTAATTAAGCC	ATTAATTA	PMID: 20389279
**SIX6**	SINE	4XRS:A	40	MEIS1	Human	TALE	CAATTATCCTGTCAA	AGGTATCA	PMID: 26550823
**SHOX**	PRD	3CMY:A	70.49	PAX3	Human	PRD	TGTAATCGATTATG	CTAATTAG	PMID: 19199574
**VAX1**	ANTP/NKL	1IG7:A	50	MSX1	Mouse	ANTP/NKL	TCCTTCAATTAGTGAC	CTAATTAG	PMID: 11580277
**VENTX**	ANTP/NKL	3RKQ:A	43.1	NKX2-5	Human	ANTP/NKL	TCAAGAGGCCCCCACTTCA	CTAATTAG	PMID: 22849347
