# Supplementary material for: DNA binding analysis of rare variants in homeodomains reveals homeodomain specificity-determining residues
Source: Nat Commun. 2024 Apr 10;15:3110. doi: 10.1038/s41467-024-47396-0 (PMC11006913; doi:10.1038/s41467-024-47396-0)
Supplement: Supplementary file 3 — Description of Additional Supplementary Files [file 41467_2024_47396_MOESM3_ESM.pdf]

Description of Additional Supplementary Files

File Name: Supplementary Data 1
Description: HD missense variants in gnomAD or ClinVar databases.

File Name: Supplementary Data 2
Description: HD alleles analyzed in this study and result “calls”.

File Name: Supplementary Data 3
Description: PBM experiment conditions, clone sequences and sequences of primers used in site-directed mutagenesis.

File Name: Supplementary Data 4
Description: Affinity and specificity scores for all 8mers for all HD alleles. [96]

File Name: Supplementary Data 5
Description: Scores from variant interpretation tools for HD variants. This table lists prediction scores of 85 HD variants instead of 92 HD variants (data unavailable for 7 variants) sourced from dbNSFP v.4.3.
