# Supplementary material for: DNA binding analysis of rare variants in homeodomains reveals homeodomain specificity-determining residues
Source: Nat Commun. 2024 Apr 10;15:3110. doi: 10.1038/s41467-024-47396-0 (PMC11006913; doi:10.1038/s41467-024-47396-0)
Supplement: Supplementary file 9 — Reporting Summary [file 41467_2024_47396_MOESM9_ESM.pdf]

nature portfolio

Corresponding author(s): ML Bulyk

Last updated by author(s): Mar 5 2024

Reporting Summary

Nature Portfolio wishes to improve the reproducibility of the work that we publish. This form provides structure for consistency and transparency in reporting. For further information on Nature Portfolio policies, see our Editorial Policies and the Editorial Policy Checklist.

Statistics

For all statistical analyses, confirm that the following items are present in the figure legend, table legend, main text, or Methods section.

n/a	Confirmed	
☐	☒	The exact sample size (*n*) for each experimental group/condition, given as a discrete number and unit of measurement
☐	☒	A statement on whether measurements were taken from distinct samples or whether the same sample was measured repeatedly
☐	☒	The statistical test(s) used AND whether they are one- or two-sided<br>*Only common tests should be described solely by name; describe more complex techniques in the Methods section.*
☐	☒	A description of all covariates tested
☐	☒	A description of any assumptions or corrections, such as tests of normality and adjustment for multiple comparisons
☐	☒	A full description of the statistical parameters including central tendency (e.g. means) or other basic estimates (e.g. regression coefficient) AND variation (e.g. standard deviation) or associated estimates of uncertainty (e.g. confidence intervals)
☐	☒	For null hypothesis testing, the test statistic (e.g. *F*, *t*, *r*) with confidence intervals, effect sizes, degrees of freedom and *P* value noted<br>*Give P values as exact values whenever suitable.*
☒	☐	For Bayesian analysis, information on the choice of priors and Markov chain Monte Carlo settings
☒	☐	For hierarchical and complex designs, identification of the appropriate level for tests and full reporting of outcomes
☒	☐	Estimates of effect sizes (e.g. Cohen's *d*, Pearson's *r*), indicating how they were calculated

Our web collection on statistics for biologists contains articles on many of the points above.

Software and code

Policy information about availability of computer code

Data collection	GenePix Pro 7
Data analysis	hmmscan version 3.2.1, GenePix Pro7, PBM Analysis Suite, R 4.3.0, the upbm custom R package, RosettaCM, Bowtie 2, MACS2

For manuscripts utilizing custom algorithms or software that are central to the research but not yet described in published literature, software must be made available to editors and reviewers. We strongly encourage code deposition in a community repository (e.g. GitHub). See the Nature Portfolio guidelines for submitting code & software for further information.

Data

Policy information about availability of data

All manuscripts must include a data availability statement. This statement should provide the following information, where applicable:

- Accession codes, unique identifiers, or web links for publicly available datasets
- A description of any restrictions on data availability
- For clinical datasets or third party data, please ensure that the statement adheres to our policy

PBM data have been deposited in the GEO database under accession number GSE233827 and have been released for public access. Other data analyzed for this study are previously published and publicly available and are listed in Methods with their Accession numbers and database links.

Research involving human participants, their data, or biological material

Policy information about studies with human participants or human data. See also policy information about sex, gender (identity/presentation), and sexual orientation and race, ethnicity and racism.

Reporting on sex and gender	No human participants. Sex was not considered in our study design. Protein binding microarrays were performed for reference and variant transcription factors regardless of the sex or gender of the individuals in which the variants were found. Sex of the donor was not considered when curating available ChIP data; both samples (human donor retina and mouse embryonic fibroblast cell line NIH-3T3) were from male donors. The research findings of our study do not apply to only one sex or gender.
Reporting on race, ethnicity, or other socially relevant groupings	No human participants.
Population characteristics	No human participants.
Recruitment	No human participants.
Ethics oversight	No human participants.

Note that full information on the approval of the study protocol must also be provided in the manuscript.

Field-specific reporting

Please select the one below that is the best fit for your research. If you are not sure, read the appropriate sections before making your selection.

☒ Life sciences ☐ Behavioural & social sciences ☐ Ecological, evolutionary & environmental sciences

For a reference copy of the document with all sections, see nature.com/documents/nr-reporting-summary-flat.pdf

Life sciences study design

All studies must disclose on these points even when the disclosure is negative.

Sample size	PBM experiments inherently measure 8mer binding at ≥16 sequences per 8mer. Each PBM experiment (variant and reference) was performed in duplicate; duplicates were chosen after analysis of reference-vs-reference comparisons to describe a baseline of detectable difference.
Data exclusions	Allelic replicates with low relative signal (as compared to other replicates) were deemed as lower-quality experiments and therefore filtered out at this step. This was assessed by comparing the upper-tail width (90th to 99th percentile) of the distribution of probe intensities for reference TF DBD samples in each allelic replicate.<br><br>We manually reviewed the differential specificity MA plots and removed one variant call identified by the edit distance criteria (ARX-R332H) which appeared to be a false positive due to the B-spline fit for this specific variant.
Replication	Robustness of replicates was assessed by analysis of reference-vs-reference comparisons (duplicate experiments from quadruplicate or higher order replicate experiments) as described above and in Methods.
Randomization	Variants and their reference controls were assigned to arrays as they became available; variant and corresponding reference are always expressed, quantified, assayed, and analyzed in parallel.
Blinding	Data were not collected blind. Data are collected systematically for all chambers in an array (including variants and corresponding reference experiments) simultaneously and analyzed in parallel. The algorithm for choosing which scans to use is described in the Methods.

Reporting for specific materials, systems and methods

We require information from authors about some types of materials, experimental systems and methods used in many studies. Here, indicate whether each material, system or method listed is relevant to your study. If you are not sure if a list item applies to your research, read the appropriate section before selecting a response.

Materials & experimental systems

n/a	Involved in the study
☐	☒ Antibodies
☒	☐ Eukaryotic cell lines
☒	☐ Palaeontology and archaeology
☒	☐ Animals and other organisms
☒	☐ Clinical data
☒	☐ Dual use research of concern
☒	☐ Plants

Methods

n/a	Involved in the study
☒	☐ ChIP-seq
☒	☐ Flow cytometry
☒	☐ MRI-based neuroimaging

Antibodies

Antibodies used

Primary rabbit anti-GST polyclonal antibody (Sigma, G7781) (1:160,000) and secondary goat horseradish peroxidase-conjugated anti-rabbit IgG monoclonal antibody (Pierce, 31460) (1:200,000) were used for Western blotting. Anti-glutathione S-transferase, rabbit IgG fraction, Alexa Fluor 488 conjugate (Invitrogen, cat. no. A11131) was used for PBM assay.

Validation

Berger, M.F. et al. Compact, universal DNA microarrays to comprehensively determine transcription-factor binding site specificities. Nat Biotechnol 24, 1429-1435 (2006).
Berger, M.F. & Bulyk, M.L. Universal protein-binding microarrays for the comprehensive characterization of the DNA-binding specificities of transcription factors. Nat Protoc 4, 393-411 (2009).
